# Five-year publication rate of podium presentations at SICOT Annual Conference: an observational study and new objective proposal of conference power

**DOI:** 10.1051/sicotj/2017019

**Published:** 2017-05-17

**Authors:** Khalid Al-Hourani, Rami Al-Aref, Robert Ley-Greaves, Fatima Ballout, Addisu Mesfin

**Affiliations:** 1 Department of Orthopaedic Surgery, Bristol Royal Infirmary Upper Maudlin Street BS2 8HW Bristol UK; 2 Stamford Hospital Columbia University CT 06902 USA; 3 Rush University Medical Center Chicago IL 60612 USA; 4 Department of Orthopaedic Surgery, University of Rochester Medical Center Rochester NY 14618 USA

**Keywords:** Conference, Publication, Rate, SICOT, Podium, Presentation

## Abstract

*Introduction*: The SICOT Conference Committee continually tries to improve the quality of presentations at their annual international meetings. However to the author’s best knowledge, no previous study has been undertaken to determine abstract quality. This study aimed to determine the five-year publication rate of presentations made at the 2009 SICOT Annual International Conference (AIC), recognise predictors of full-text publication, identify inconsistencies between presentations and publications, and determine presentation-publication delay.

*Methods*: We retrieved all 329 oral presentation abstracts from the 2009 SICOT Conference, recorded fundamental study details and conducted a comprehensive, electronic search of Medline and PubMed to determine publication status. For subsequent publications, we examined for inconsistencies between presentation abstracts and full-text publications, whether there were retrospectively identifiable publication predictors and calculated presentation-publication delay.

*Results*: The five-year publication rate for all presentations was 31.3%, for oral presentations. The average presentation-publication delay was 23.4 months. Observational studies were the most commonly published studies. Publications most commonly resulted from studies related to hip and knee subspecialties.

*Conclusion*: Our study shows that almost one third of all abstracts presented at SICOT led to a full-text publication. This is a positive outcome particularly when made in comparison to similar studies of other reputable international conferences such as European Federation of Orthopaedics and Traumatology (EFORT) and American Academy of Orthopaedic Surgeons (AAOS). This study re-enforces SICOT’s reputation as a world leading international conference with a strict peer-review process yielding high-quality presentations.

## Introduction

International conferences remain an excellent way of disseminating research and furthering evidence-based medicine, thereby potentially influencing clinical practice. Abstracts presented at conferences also provide a forum for debate, critical analysis and discussion, which in itself is a form of peer review. Previous studies have suggested up to 63% of major orthopaedic textbook chapters having included results from abstracts presented at international orthopaedic meetings [1]. However, abstracts in themselves are not always readily searchable postpresentation and this can lead to difficulties in citing any evidence presented at meetings. A peer-reviewed publication therefore remains the gold standard when judging the quality and validity of a study.

The International Society of Orthopaedic Surgery and Traumatology (SICOT) holds an annual international conference that provides a well-known and important forum to share novel research findings. There is therefore great emphasis on conferences such as this in providing a substantial portion of continued medical education for surgeons and allied health professionals. The SICOT Programme Committee is responsible for the selection of podium presentations as part of a strict, blinded and peer-reviewed process. This process has led to podium presentations at this meeting becoming increasingly well regarded, and the knowledge acquired as a result of these presentations is becoming more rapidly incorporated into clinical practice. For this reason, it is essential to question the validity of such podium presentations.

Our aim was therefore to: (1) evaluate the five-year publication rate of podium presentations of SICOT Annual International Conference, (2) to evaluate the consistency of abstracts presented and subsequent consistency with the full peer-reviewed publication and (3) to discuss our findings with other well-known orthopaedic international conferences.

## Materials and methods

We reviewed all podium presentations at the 2009 SICOT XXV Triennial World Conference in Prague, Czech Republic. The abstracts from the 2009 SICOT Conference were chosen in order to give at least five years for subsequent publications and as it also allows the methods to fall in line with those used in previous similar studies [1–4]. All 329 podium presentations published in the 2009 SICOT Conference podium abstract book were included in the study. Poster abstracts were excluded due to a higher scientific value being placed on podium presentations.

The Bhandari methodology previously described was the main strategy used to search for abstracts in PubMed and Medline [1]. We used two separate reviewers to search for abstracts in order to increase reliability. Any abstracts, which were not agreed upon by the two reviewers, were reviewed by the first author in order to come to a majority decision. Comparing the abstract and final publication through similarity of content provided the basis for inclusion. This only comprised a small minority of the overall number.

A sizeable database was created for data input and final analysis. Each study was given a unique identification number and a variety of data were recorded for each study including study design, methodology, statistical analysis and study outcomes. All analysis was done using GraphPad Prism statistical analysis software. A median (range) value was obtained for time to publication and number of centres involved in each study. Journal impact factors were sorted accordingly in the database to rank the highest and lowest impact factor journals in which a publication occurred. The database was sorted by journal title to provide overall journal ranking in terms of number of published abstracts. A similar method was used to rank subspecialties published, type of study, country and associated level of evidence according to the Oxford Centre for Evidence-Based Medicine grading system. A goodness-of-fit test was used to assess correlation between parameters and publication, with publication set as a binary figure of either 1 (indicating publication) or 0 (indicating no publication). Differences in study parameters such as between the presented abstract and a final publication were recorded for analysis. These parameters included methodology, study size, number of centres involved as well as primary and secondary outcomes. A difference would be described as any significant change in these parameters between abstract and final publication. Consistency was therefore measured as a mean percentage with an attributed confidence interval. If there was no eventual publication then only information on the abstract was collected.

## Results

Three hundred and twenty-nine abstracts were podium presentations at the 2009 International Society of Orthopaedics and Traumatology (SICOT). From these, 103 (31.3%) were published with a complete report with a median time to publication of 23.4 months (0–72 months). The 103 papers were published in 45 distinct journals. The most commonly published subspecialties were paediatric orthopaedics, hip and knee arthroplasty. A breakdown of the top 10 journals in which these papers were published and their respective impact factors are shown in Table 1. The median (range) impact factor score of the journals, which published the podium research, was 1.730 (0.181–19.967). The journal, in which an abstract was published, with the highest impact factor was The British Medical Journal (impact factor 19.967), with the lowest impact factor journal being The European Journal of Orthopaedic Surgery and Traumatology (impact factor 0.181).

**Table 1.** Showing the top 10 journals in rank order of papers published.

Ranking (number published)	Journal	Impact factor
1 (9)	International Orthopaedics	2.387
2 (7)	Journal of the Medical Association of Thailand	0.314
3 (5)	Journal of Orthopaedics and Traumatology	1.777
4 (5)	Journal of Foot and Ankle Surgery	0.845
5 (5)	Indian Journal of Orthopaedics	0.64
6 (5)	Acta Orthopaedics Belgica	0.629
7 (4)	Clinical Orthopaedics & Related Research	2.765
8 (4)	Orthopaedics	1.256
9 (3)	Spine	2.078
10 (3)	Journal of Paediatric Orthopaedics	1.163

## Characteristics of abstracts published

The majority of published abstracts were submitted from single centre institutions with a median of 1.12 (1–3) with some multicentric publications. Sample sizes ranged from a minimum of two to a maximum of 15 646 with a median of 43. The largest number of studies submitted to SICOT was from India with 12.2%, while Thailand was the most published country with 16.0% of all completed publications (Tables 2 and 3, respectively). Moreover, the United States had the highest ratio of submitted to publish with a ratio of 63.6% (11 submitted to SICOT and seven publications achieved). Interestingly, both sample size and subspecialty of the abstracts submitted were not significant when tested using chi-squared goodness of fit (*p* > 0.05) and therefore likely played no role in successful publication of the submitted abstract.

**Table 2.** Showing the top five countries with presented oral abstracts. Total *N* = 329.

Ranking	Country	Number (%)
1	India	40 (12)
2	Thailand	36 (11)
3	Russia	30 (9)
4	Japan	22 (7)
5	Korea	21 (6)

**Table 3.** Showing the top five countries with abstracts proceeding to full publication. Total *N* = 103.

Ranking	Country	Number (%)
1	Thailand	17 (16)
2	India	13 (13)
3	France	9 (9)
4	United States of America	7 (7)
5	United Kingdom	6 (6)

## Study methodology

We found that the dominant research design was observational comprising 81.8% of all submitted papers. Moreover, of the abstracts that were eventually published, Retrospective observational studies were also the primary research design accounting for 77.7% of all published papers and these were all level III evidence. Randomised control trials (level I evidence) consisted of only 1.5% and 1.1% of submitted and published articles, respectively. Of the presented abstracts, 78.4% mentioned clear statistical methods, while 88.3% of the abstracts that were published contained clear statistical methods signifying a potential benefactor in abstracts that proceed to full publication. The median (range) level of evidence of all studies was 3 (1–5) which corresponds to most studies being a mix of prospective and retrospective.

## Consistency of abstracts with final publication

We identified seven major categories to compare the consistency between the submitted abstract and the eventual completed full publication. These categories include precision variances, change in hypothesis/study objective focus, differing study design, variation in sample size, changes in statistical analysis, and differing primary and secondary outcomes. The most consistent categories were the hypothesis and study design categories with mean consistency of 90.4% (CI 82.6%, 95.5%) and 88.3% (CI 80.0%, 94.0%), respectively. The least consistent category was sample size with a mean of 69.2% (CI 58.8%, 78.3%). Precision studies and statistical analysis remained fairly consistent with means of 81.9% (CI 72.6%, 89.1%) and 77.7% (CI 67.9%, 85.6%), respectively. Lastly primary and secondary outcomes also remained consistent with corresponding means of 87.2% (CI 78.8%, 93.2%) and 85.1% (CI 76.3%, 91.6%).

## Discussion

The International Society of Orthopaedics and Traumatology (SICOT) is a highly regarded conference that is dedicated to the discussion and dissemination of all orthopaedic topics. Since its initiation in 1929 it has had 37 annual international conferences. Podium and paper presentations undergo a strict peer-review process and are considered high quality, however it is important to analyse this objectively through publication rates as this represents a permanent, accessible and validated representation of the scientific abstract.

Rates of full publication of presented abstracts range widely in the literature from 11% to 78% [1, 2, 8, 9]. Our study is the first to analyse the SICOT Annual International Conference. We have demonstrated a five-year publication rate of 31.3%, which is consistent with other high profile conferences such as AAOS (34%). Furthermore of those published, we reported mean time to publication of 23.4 months, which falls in line with other notable conferences such as AAOS and EFORT.

It is reassuring to note such a high consistency between abstract and full publication with regard to study hypothesis and study designs which is a direct reflection of aims and methods. With regard to the least consistent category of study sample size, this is often owed to the fact that studies are amended following feedback from audience members’ postpresentation [10]. Observational studies remain the most popular category of study, however, there remains a clear lack of level 1 randomised control trials presented at this meeting. Future work should include re-analysing more recent conferences to monitor any trends.

There are a number of reasons why presented abstracts may not achieve full publication. This includes failure to submit as a full article, pessimism about chances of an article being accepted and rejected by journals through the peer-review process. It is also likely that we have underestimated the publication rate of SICOT as we conducted a PubMed and Medline search and therefore publications not indexed in these databases would be missed. However, these remain highly popular databases for research articles and therefore are the databases most likely to reach the majority of our audience. One further possible limitation is that we have limited our search to a five-year period after presentation in 2009. While the authors recognise some studies could be published after five years, there is evidence to suggest that the majority of papers are published within four years of presentation [5–7].

To conclude, this is the first study to analyse this world renowned international conference and has demonstrated results comparable with those of other notable conferences of a similar standard. Since 2009, annual submissions to SICOT have steadily increased and we would expect podium presentations to be of an even higher scientific value and subsequently an even higher publication rate in recent years. The authors propose another analysis to confirm this hypothesis and we propose a new objective conference grading system based on the publication rate.

## Compliance with ethical standards

### Conflict of interest

On behalf of all authors, the corresponding author states that there is no conflict of interest.

### Funding

There is no funding source.

### Ethical approval

This article does not contain any studies with human participants or animals performed by any of the authors.

### Informed consent

Not applicable.
